# Association between erythrocyte parameters and metabolic syndrome in urban Han Chinese: a longitudinal cohort study

**DOI:** 10.1186/1471-2458-13-989

**Published:** 2013-10-21

**Authors:** Shuo Wu, Haiyan Lin, Chengqi Zhang, Qian Zhang, Dongzhi Zhang, Yongyuan Zhang, Wenjia Meng, Zhenxin Zhu, Fang Tang, Fuzhong Xue, Yanxun Liu

**Affiliations:** 1 Department of Epidemiology and Biostatistics, School of Public Health, Shandong University, Jinan 250012, China; 2 Health Management Center, Shandong Provincial QianFoShan Hospital, Jinan 250014, China; 3 Center for Health Management, Provincial Hospital affiliated to Shandong University, Jinan 250012, China

**Keywords:** Metabolic syndrome (MetS), Erythrocyte parameters, Longitudinal cohort study, Generalized estimated equation (GEE)

## Abstract

**Background:**

Although various cross-sectional studies have shown that erythrocyte parameters, including red blood cell (RBC), hemoglobin (Hb) and hematocrit (HCT), were linked with metabolic syndrome (MetS), few longitudinal studies have been used to confirm their relationship. The study, therefore, constructed a large-scale longitudinal cohort in urban Chinese population to highlight and confirm the association between erythrocyte parameters and MetS/its components.

**Methods:**

A longitudinal cohort with 6,453 participants was established based on the routine health check-up systems to follow up MetS, and Generalized Estimating Equation (GEE) model was used to detect the association between erythrocyte parameters and MetS/its components (obesity, hyperglycemia, dyslipidemia, and hypertension).

**Results:**

287 MetS occurred over the four-year follow-up, leading to a total incidence density of 14.19 per 1,000 person-years (287/20218 person-years). Both RBC and Hb were strongly associated with MetS (RR/95% CI, P value; 3.016/1.525-5.967, 0.002 for RBC; 3.008/1.481-6.109, 0.002 for Hb), with their dose–response trends detected. All three erythrocyte parameters (RBC, Hb and HCT) were found to be associated with obesity, hypertension and dyslipidemia with similar dose–response trends respectively, while only Hb showed a significant association with hyperglycemia.

**Conclusions:**

Elevated erythrocyte parameters were confirmed to be associated with MetS/its components in urban Chinese population, suggesting that erythrocyte parameters might be served as a potential predictor for risk of MetS.

## Background

The metabolic syndrome (MetS) is characterized by obesity, hyperglycemia, dyslipidemia, hypertension and insulin resistance (IR) [1,2]. Various cross-sectional studies have demonstrated that erythrocyte parameters, including red blood cell (RBC), hemoglobin (Hb) and hematocrit (HCT), were associated with MetS [2-8]. These cross-sectional studies showed that elevated RBC was associated with MetS in Taiwan [2], Israel [3], Korea [4], Japan [5,6], Hb in Thailand [7] and Japan [6], HCT in Thailand [7] and Japan [6,8]. These positive associations were further detected in an Ethiopian cohort [9]. Furthermore, in a Japanese cohort [10], HCT was reported to be positively associated with insulin resistance, which is the basic pathogenesis for MetS. As most current results were reported from cross-sectional studies, and few from cohort studies, further longitudinal cohort studies are required to confirm the assumption in different populations.

The study, we established a longitudinal cohort with 6,453 participants based on the routine health check-up systems in urban Chinese population to follow up MetS, and each individual in this cohort was undergone at least three repeated health checks in the five years (January 2005 to January 2010). Furthermore, Generalized Estimating Equation (GEE) model, which could handle the repeat measurement data with high autocorrelation in the framework of logistic regression model [11-14], was used to detect the association between erythrocyte parameters and MetS/its components (obesity, hyperglycemia, dyslipidemia, and hypertension).

## Methods

### Study population

**Figure 1:**
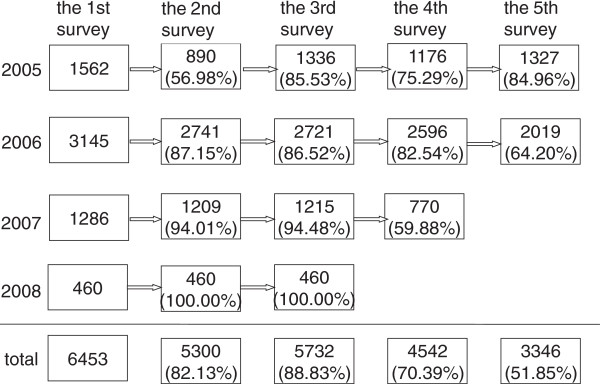
The samples of repeated surveys at each year.

A large scale longitudinal cohort was set up in 2005 on middle-to-upper class urban Han Chinese who attended routine health check-up at the Centers for Health Management of Shandong Provincial Hospital and Shandong Provincial Qianfoshan Hospital. Four groups of participants without cerebral infarction, cardiovascular disease, coronary artery bypass surgery, MetS and its single component in their first check-up at the year of 2005, 2006, 2007 and 2008 were included in the baseline of our longitudinal cohort study respectively. Figure 1 showed the total of 6453 participants having at least three repeated health check-up within five years (January 2005 to January 2010), and the samples of repeated surveys each year.

All individuals in the longitudinal cohort underwent a general health questionnaire, anthropometric measurements, and laboratory tests. The general health questionnaire covered the current status of smoking, alcohol intake, diet, sleeping quality and physical activity. Anthropometric measurements involved height, weight, and blood pressure. Both height and weight were measured with light clothing without shoes. Body mass index (BMI) was calculated as weight/height^2^ (kg/m^2^) as an evidence of obesity. Blood pressure was measured using Omron HEM-907 by the cuff-oscillometric method on the right arm in sitting position after a 5-min rest, and the mean systolic and diastolic blood pressure values of two measurements were recorded respectively. While the participant was fasting, a venous blood sample was taken for laboratory test. Laboratory tests included RBC, Hb, HCT, white blood count (WBC), platelet distribution width (PDW), mean platelet volume (MPV), thrombocytocrit (PCT), glucose, total cholesterol (CHOL), low-density lipoprotein (LDL), high-density lipoprotein (HDL), triglycerides (TG), gamma-glutamyl transpeptidase (GGT), serum albumin (ALB), serum globulins (GLO), blood urea nitrogen (BUN), and serum creatinine (SCr), etc. This study was approved by the Ethics Committee of School of Public Health, Shandong University, and all participants were given informed written consent.

### Definition of the metabolic syndrome

Considering the target population was Chinese with their specific physiological characteristics, Diabetes Branch of the Chinese Medical Association (CDS) [15] was used as the MetS diagnostic criteria, which is very popular for the Chinese population in clinical practice. MetS was defined as presence of three or more of the following four medical conditions: 1) overweight or obesity, i.e. BMI ≥25.0 Kg/m^2^; 2) hypertension, i.e. systolic blood pressure (SBC) ≥140 mmHg, or diastolic blood pressure (DBP) ≥90 mmHg, or previously diagnosed; 3) dyslipidemia, i.e., fasting TG ≥1.7 mmol/L, or fasting HDL <0.9 mmol/L; 4) hyperglycemia, i.e. fasting blood-glucose (FPG) ≥6.1 mmol/L, or 2 h Post-meal Glucose (PG) ≥7.8 mmol/L, or previously diagnosed.

### Statistical analysis

To account for missing values, multiple imputation was performed. Since imputation method was depended on the patterns of the missing data and the types of the imputed variables, without loss of generality, the Markov chain Monte Carlo (MCMC) method was chosen according to MI Procedure of SAS 9.1.3 [16]. Most variables had less than 2% missing observations before imputation except diet, drinking, smoking, quality of sleep and physical activity having less than 10% missing values. The original continuous erythrocyte parameters were categorized into 4 levels (Q1-Q4) using the 3 quartiles of P25, P50 and P75 as critical values, with ≤ P25 for Q1, >P25 and ≤ P50 for Q2, >P50 and ≤ P75 for Q3, and > P75 for Q4 respectively. Summary statistics were used to illustrate the distribution characteristics for variables of interest at each repeated surveys, and student's *t* test for continuous variables and chi-square test for categorical variables were used to detect the statistical significances compared with the first survey (baseline). As GEE model could handle the repeat measurement data with high autocorrelation in the framework of logistic regression model [11-14], it was used to detect the association between erythrocyte parameters and MetS/its components. Simple GEE model was firstly used to select variables associated with MetS/its components, then variables which were significant at the level of 0.05 in the simple GEE analysis entered the multiple GEE model to adjust the potential confounding. The 'Logit' link function was chosen in GEE analysis, with significance level 0.05. All the statistical analyses were performed on SAS 9.1.3.

## Results

Table 1 summarized the characteristics of erythrocyte parameters levels and other potential confounding factors of the participants at each repeated survey, which showed that most factors were generally higher than that in the first survey (baseline). A total of 294 cases of MetS occurred over the four-year follow-up, leading to a total incidence density of 14.19 per 1,000 person-years (287/20218 person-years). During the follow up, 3 participants were diagnosed as cerebral infarction (person-years), 141 participants were diagnosed as cardiovascular disease (person-years) and no-one underwent coronary artery bypass surgery (see Additional file 1: Table S20).

**Table 1:** Distribution of erythrocyte parameters and other potential confounding factors

**Variables**	**The 1st survey (N = 6453)**	**The 2nd survey (N = 5300)**	**The 3rd survey (N = 5732)**	**The 4th surveys (N = 4542)**	**The 5th survey (N = 3346)**
**age**	38.563 ± 11.444	39.798 ± 11.572	40.619 ± 11.406^*^	42.413 ± 11.495^*^	43.317 ± 11.352^*^
**sex**					
**male**	2688	2196	2415	1861	1351
**female**	3765	3104	3317	2681	1995
**RBC**	4.769 ± 0.456	4.705 ± 0.454^*^	4.698 ± 0.454^*^	4.678 ± 0.463^*^	4.7 ± 0.432^*^
**Hb**	142.454 ± 14.885	142.845 ± 15.27	141.73 ± 15.169^*^	142.569 ± 15.381	142.115 ± 16.465
**HCT**	42.888 ± 3.905	42.408 ± 3.951^*^	42.015 ± 3.889^*^	42.191 ± 3.964^*^	41.846 ± 3.869^*^
**GGT**	18.004 ± 16.393	20.016 ± 20.032^*^	19.383 ± 16.419^*^	21.08 ± 20.193^*^	21.336 ± 20.323^*^
**ALB**	46.575 ± 2.427	45.789 ± 2.784^*^	45.362 ± 2.742^*^	45.128 ± 2.69^*^	44.991 ± 2.436^*^
**GLO**	27.016 ± 3.838	27.133 ± 3.973	28.253 ± 3.972^*^	29.128 ± 4.046^*^	30.314 ± 3.826^*^
**BUN**	4.746 ± 1.197	4.698 ± 1.175^*^	4.664 ± 1.154^*^	4.772 ± 1.204	4.812 ± 1.145^*^
**S-Cr**	76.943 ± 13.736	77.897 ± 13.842^*^	77.502 ± 14.072^*^	78.587 ± 14.694^*^	77.012 ± 12.743
**WBC**	6.167 ± 1.466	6.069 ± 1.483^*^	6.054 ± 1.479^*^	6.142 ± 1.501	6.182 ± 1.489
**PDW**	12.346 ± 1.712	12.323 ± 1.722	12.226 ± 1.671^*^	12.164 ± 1.669^*^	12.088 ± 1.63^*^
**MPV**	10.454 ± 0.811	10.448 ± 0.946	10.42 ± 0.803^*^	10.398 ± 0.802^*^	10.428 ± 0.796
**PCT**	0.247 ± 0.089	0.257 ± 0.320^*^	0.254 ± 0.266^*^	0.249 ± 0.055	0.245 ± 0.054
**Diet**					
**Vegetarian**	3454	2194^*^	2098^*^	1906^*^	1214^*^
**normal**	1863	792	750	550	463
**meat-based**	1118	2283	2866	2064	1653
**sea food**	18	31	18	22	16
**Drinking**					
**no**	3698	2959	3333	2731^*^	1977
**yes**	2755	2341	2399	1811	1369
**Smoking**					
**no**	5274	4323	4678	3726	2718
**yes**	1179	977	1054	816	628
**Sleep**					
**≥fair**	6270	5173	5557	4373^*^	3250
**<fair**	183	127	175	169	96
**Exercise**					
**never/seldom**	4694	3902	4267^*^	3469^*^	2477
**often**	1759	1398	1465	1073	869

*P < 0.05 compared with the first survey (baseline);

The abbreviations of the variables: RBC = red blood cell; Hb = Hemoglobin; HCT = Hematocrit; GGT = gamma-glutamyl transpeptidase; ALB = serum albumin; GLO = serum globulins; BUN = blood urea nitrogen; S-Cr = serum creatinine; WBC = white blood cell; PDW = Platelet distribution width; MPV = mean platele volume; PCT = Thrombocytocrit; Diet: 0: Vegetarian, 1: normal, 2: meat-based 3: sea food (the major kinds of food used to have); Drinking: 0: never, 1: seldom, 2: often, wine, 3: often beer, 4: often, Chinese spirits, 5: often, mixed all kinds; Smoking: 0: never, 1: seldom, 2: quit, 3: 1-4/d, 4: 5 -15/d, 5: >15/d; Quality of sleep: 0: excellent, 1: well, 2: fair 3: poor, 4: very poor (evaluated by themselves); Physical activity 0: never, 1: seldom (1–2 times a week), 2: often or everyday (more than 3 times a week).

Table 2 showed the selected variables associated with MetS at α = 0.05 level. It indicated that each of the 3 erythrocyte parameters (RBC, Hb, and HTC) with 9 potential confounding factors, including gender, age, GGT, GLO, BUN, WBC, diet, drinking and smoking might be linked with MetS. Also, each of the 3 erythrocyte parameters might be linked with the four components of MetS with their specific potential confounding factors respectively (see Additional file 2: Table S1, Additional file 3: Table S2, Additional file 4: Table S3 and Additional file 5: Table S4 for details).

**Table 2:** The association analyses result from simple GEE model (MetS as dependent variable)

**Quartiles**	**Estimate**	**ERR**	**Z**	**P > |Z|**	**RR**	**Lower 95% confidence limits**	**Upper 95% confidence limits**
**red blood cell**							
**Q4**	1.284	0.244	5.271	<0.001	3.612	2.241	5.824
**Q3**	0.602	0.261	2.308	0.021	1.825	1.095	3.042
**Q2**	0.050	0.292	0.170	0.865	1.051	0.593	1.864
**Q1**	ref	ref	ref	ref	ref	ref	ref
**hemoglobin**							
**Q4**	1.271	0.209	6.078	<0.001	3.564	2.366	5.370
**Q3**	0.492	0.232	2.118	0.034	1.635	1.037	2.577
**Q2**	0.251	0.240	1.045	0.296	1.285	0.803	2.056
**Q1**	ref	ref	ref	ref	ref	ref	ref
**hematocrit**							
**Q4**	1.005	0.210	4.776	<0.001	2.732	1.809	4.127
**Q3**	0.406	0.234	1.736	0.083	1.500	0.949	2.371
**Q2**	0.123	0.249	0.496	0.620	1.131	0.694	1.844
**Q1**	ref	ref	ref	ref	ref	ref	ref
**gender**	−0.946	0.157	−6.034	<0.001	0.388	0.286	0.528
**age**	0.410	0.039	10.596	<0.001	1.507	1.397	1.625
**GGT**	0.012	0.002	7.235	<0.001	1.012	1.009	1.016
**ALB**	−0.050	0.025	−1.981	0.048	0.951	0.906	0.999
**GLO**	0.072	0.015	4.863	<0.001	1.074	1.044	1.106
**BUN**	0.177	0.049	3.599	<0.001	1.193	1.084	1.314
**S-Cr**	0.010	0.006	1.668	0.095	1.010	0.998	1.022
**WBC**	0.284	0.031	9.061	<0.001	1.328	1.249	1.412
**PDW**	0.005	0.042	0.119	0.905	1.005	0.926	1.091
**MPV**	−0.093	0.094	−0.983	0.326	0.912	0.758	1.096
**PCT**	−0.588	1.229	−0.478	0.632	0.555	0.050	6.176
**Diet**	0.223	0.073	3.049	0.002	1.250	1.083	1.442
**Drinking**	0.173	0.046	3.796	0.001	1.189	1.087	1.300
**Smoking**	0.143	0.044	3.246	0.001	1.153	1.058	1.257
**Sleep**	0.109	0.076	1.432	0.152	1.115	0.961	1.294
**Exercise**	−0.013	0.159	−0.080	0.937	0.987	0.723	1.348

Table 3 illustrated the summarized results of the association analyses between erythrocyte parameters and MetS/its components after adjusting potential confounding factors by multiple GEE model. (Confounding variables were not shown, detailed information seeing Additional file 6: Table S5, Additional file 7: Tables S6, Additional file 8: Table S7, Additional file 9: Table S8, Additional file 10: Table S9, Additional file 11: Table S10, Additional file 12: Table S11, Additional file 13: Table S12, Additional file 14: Table S13, Additional file 15: Table S14, Additional file 16: Table S15, Additional file 17: Table S16, Additional file 18: Table S17, Additional file 19: Table S18 and Additional file 20: Table S19.). It revealed that the top quartiles (Q4) of RBC and Hb were strongly associated with MetS (RR/95%CI, P value; 3.016/1.525-5.967, 0.002 for RBC; 3.008/1.481-6.109, 0.002 for Hb) using Q1 as reference level. Although no significant for Q2 and Q3, trends of increased of RR were observed from Q2 to Q4, indicating that there were dose–response trends between the 2 erythrocyte parameters and MetS. In addition, all three erythrocyte parameters (RBC, Hb and HCT) were found to be associated with obesity, hypertension and dyslipidemia with similar dose–response trends respectively, while only Hb showed a significant association with hyperglycemia.

**Table 3:** The summary results of the association analyses between erythrocyte parameters and MetS/its components after adjusting potential factors by multiple GEE model

**Parameters**		**MetS**			**Obesity**			**Hyperglycemia**			**Hypertension**			**Dyslipdemia**		
		**Estimate**	**P > |Z|**	**RR**	**Estimate**	**P > |Z|**	**RR**	**Estimate**	**P > |Z|**	**RR**	**Estimate**	**P > |Z|**	**RR**	**Estimate**	**P > |Z|**	**RR**
**Red blood cell**	Q4	1.104	0.002	3.016	0.474	<0.001	1.606	0.215	0.202	1.239	0.484	0.001	1.622	0.431	<0.001	1.539
	Q3	0.523	0.081	1.688	0.364	0.001	1.439	0.075	0.606	1.078	0.271	0.042	1.312	0.251	0.002	1.285
	Q2	−0.039	0.895	0.961	0.006	0.955	1.006	0.068	0.605	1.071	0.113	0.389	1.120	0.123	0.103	1.131
	Q1	ref	ref	ref	ref	ref	ref	ref	ref	ref	ref	ref	ref	ref	ref	ref
**Hemolglobin**	Q4	1.101	0.002	3.008	0.693	<0.001	2.000	0.654	0.001	1.923	0.749	<0.001	2.114	0.536	<0.001	1.709
	Q3	0.454	0.144	1.575	0.447	<0.001	1.564	0.519	0.001	1.680	0.390	0.006	1.477	0.254	0.003	1.289
	Q2	0.340	0.162	1.405	0.387	<0.001	1.473	0.307	0.025	1.359	0.273	0.035	1.314	0.082	0.251	1.086
	Q1	ref	ref	ref	ref	ref	ref	ref	ref	ref	ref	ref	ref	ref	ref	ref
**Hemocrite**	Q4	0.583	0.079	1.792	0.362	0.009	1.436	0.028	0.874	1.029	0.368	0.021	1.445	0.257	0.009	1.293
	Q3	0.214	0.479	1.238	0.297	0.013	1.346	0.151	0.320	1.163	0.324	0.018	1.383	0.195	0.018	1.215
	Q2	0.034	0.892	1.035	0.220	0.028	1.246	0.014	0.915	1.014	0.149	0.250	1.161	0.003	0.969	1.003
	Q1	ref	ref	ref	ref	ref	ref	ref	ref	ref	ref	ref	ref	ref	ref	ref

## Discussions

This study mainly attempted to confirm the association between erythrocyte parameters and MetS/its components using the longitudinal cohort. Although the longitudinal cohort study was based on routine health check-up in urban Han Chinese population from middle to upper socioeconomic strata, the positive associations between erythrocyte parameters and MetS/its components were observed, which were also detected in other two cohort study [9].

RBC had been reported to be associated with MetS in various populations by cross-sectional studies [2-6] and in Ethiopia by a cohort studies [9]. At present study, we not only confirmed that RBC was associated with MetS and its 3 single components (dyslipidemia, obesity and hypertension), but also observed the dose–response trends (seeing Table 3 or Additional file 7: Table S6, Additional file 8: Tables S7 and Additional file 9: Table S8). These results highlighted the positive association between RBC and MetS. In pathogenesis, this linkage might be explained by the insulin resistance(IR) mechanisms in the development of MetS, because insulin and insulin growth factors I and II supporting erythropoiesis in both vitro [17-21] and vivo [22-24] had been detected in laboratory studies.

As another important erythrocyte parameter, Hb also had been reported to be associated with MetS in Thailand [7] and Japan [6] by Cross-sectional studies, as well as in Ethiopia [9] by a cohort study. This positive association was also detected in our longitudinal cohort study with a potential dose–response trend between them (seeing Table 3 or Additional file 11: Table S10, Additional file 12: Table S11, Additional file 13: Table S12, Additional file 14: Table S13 and Additional file 15: Table S14). The possible mechanism might be supported by the following pathogenesis. Hb is a well recognized carrier and buffer of nitric oxide (NO), and various compounds of Hb with NO can affect Hb-oxygen affinity of the whole blood [25]. Disturbed NO synthesis may exert an adverse effect on endothelial dysfunction through the L-arginine-NO pathway [26]. Furthermore, endothelial dysfunction was reported to be associated with MetS [27,28]. All these evidences expect the association between Hb and MetS/its components in population level.

Elevated HCT could increase blood viscosity and peripheral resistance to blood flow, and further contribute to IR [29-31]. Therefore, the association between HCT and MetS/its components should be observed in population level. In this paper, HCT associating with obesity, hypertension and dyslipdemia were all detected in urban Han Chinese population (seeing Table 3 or Additional file 17: Table S16, Additional file 18: Table S17, Additional file 19: Table S18), while no statistical significant association between HCT and MetS/ hyperglycemia was found (seeing Table 3, Additional file 16: Table S15 or Additional file 20: Table S19). Similar results were also observed in Ethiopia [9] and Japan [9] by cohort studies, as well as in Thailand [7] and Japan [9,11] by Cross-sectional studies.

Several limitations of this study must be considered. a) Selection bias might exist due to the samples just from routine health check-up population for middle-to-upper class urban Han Chinese. b) Owing to the absence of waist circumference measurement, the diagnostic criteria of MetS was just based on China Diabetes Federation, rather than international standard criteria. c) The medication history and menstrual history of participants might be significant confounding factors, but they were absent in our database. d) Hematological parameter categories were based on a single assessment of blood, which may cause a misclassification bias. It is, therefore, desired to conduct a perfect longitudinal cohort study in general population for further highlighting the association between erythrocyte parameters and MetS.

## Conclusion

In conclusion, elevated erythrocyte parameters were confirmed to be associated with MetS/its components in urban Chinese population, suggesting that erythrocyte parameters might be a potential predictor for risk of MetS.

## Competing interests

There is no conflict of interest for any of the authors. All authors had access to the data and were involved in drafting the article and revising it critically for important intellectual content.

## Authors’ contributions

In our work, YL, FX and CZ designed the study and directed its implementation, including quality assurance and control. FT, HL and DZ did the clinical exam and collected the data. QZ, YZ, WM and ZZ helped analyzing the data. SW participated much of the above work and led the writing of the paper. All authors read and approved the final manuscript.

## Pre-publication history

The pre-publication history for this paper can be accessed here:

http://www.biomedcentral.com/1471-2458/13/989/prepub

## Supplementary Material

Additional file 1: Table S20 The morbidity number of repeated surveys at each year. Click here for file

Additional file 2: Table S1 The association analyses result from simple GEE model (obesity as dependent variable). Click here for file

Additional file 3: Table S2 The association analyses result from simple GEE model (dyslipidemia as dependent variable). Click here for file

Additional file 4: Table S3 The association analyses result from simple GEE model (hyperglycemia as dependent variable). Click here for file

Additional file 5: Table S4 The association analyses result from simple GEE model (hypertension as dependent variable). Click here for file

Additional file 6: Table S5 Multiple GEE analysis of red blood cell and MetS after adjusting other potential confounding factors. Click here for file

Additional file 7: Table S6 Multiple GEE analysis of red blood cell and obesity after adjusting other potential confounding factors. Click here for file

Additional file 8: Table S7 Multiple GEE analysis of red blood cell and hypertension after adjusting other potential confounding factors. Click here for file

Additional file 9: Table S8 Multiple GEE analysis of red blood cell and dyslipidemia after adjusting other potential confounding factors. Click here for file

Additional file 10: Table S9 Multiple GEE analysis of red blood cell and hyperglycemia after adjusting other potential confounding factors. Click here for file

Additional file 11: Table S10 Multiple GEE analysis of hemoglobin and MetS after adjusting other potential confounding factors. Click here for file

Additional file 12: Table S11 Multiple GEE analysis of hemoglobin and **obesity** after adjusting other potential confounding factors. Click here for file

Additional file 13: Table S12 Multiple GEE analysis of hemoglobin and hypertension after adjusting other potential confounding factors. Click here for file

Additional file 14: Table S13 Multiple GEE analysis of hemoglobin and dyslipidemia after adjusting other potential confounding factors. Click here for file

Additional file 15: Table S14 Multiple GEE analysis of hemoglobin and hyperglycemia after adjusting other potential confounding factors. Click here for file

Additional file 16: Table S15 Multiple GEE analysis of hematocrit and MetS after adjusting other potential confounding factors. Click here for file

Additional file 17: Table S16 Multiple GEE analysis of hematocrit and obesity after adjusting other potential confounding factors. Click here for file

Additional file 18: Table S17 Multiple GEE analysis of hematocrit and hypertension after adjusting other potential confounding factors. Click here for file

Additional file 19: Table S18 Multiple GEE analysis of hematocrit and dyslipidemia after adjusting other potential confounding factors. Click here for file

Additional file 20: Table S19 Multiple GEE analysis of hematocrit and hyperglycemia after adjusting other potential confounding factors. Click here for file
