# Supplementary material for: Association between erythrocyte parameters and metabolic syndrome in urban Han Chinese: a longitudinal cohort study
Source: BMC Public Health. 2013 Oct 21;13:989. doi: 10.1186/1471-2458-13-989 (PMC4016498; doi:10.1186/1471-2458-13-989)
Supplement: Additional file 1: Table S20 — The morbidity number of repeated surveys at each year. [file 1471-2458-13-989-S1.doc]

**Table S20 The morbidity number of repeated surveys at each year**

| **Disease** | **the 1st survey** | **The 2nd survey** | **the 3rd survey** | **the 4th survey** | **the 5th survey** | **incidence density/ 1,000 person-years** |
| --- | --- | --- | --- | --- | --- | --- |
| **MetS** | 0 | 60 | 80 | 84 | 63 | 14.195 |
| **BMI** | 0 | 479 | 588 | 528 | 399 | 98.625 |
| **hyperglycemia** | 0 | 135 | 238 | 237 | 228 | 41.448 |
| **hypertension** | 0 | 271 | 347 | 301 | 285 | 59.551 |
| **dyslipidemia** | 0 | 993 | 1488 | 884 | 520 | 192.156 |
| **cardiovascular disease** | 0 | 35 | 40 | 38 | 28 | 6.974 |
