# Supplementary material for: Association between erythrocyte parameters and metabolic syndrome in urban Han Chinese: a longitudinal cohort study
Source: BMC Public Health. 2013 Oct 21;13:989. doi: 10.1186/1471-2458-13-989 (PMC4016498; doi:10.1186/1471-2458-13-989)
Supplement: Additional file 2: Table S1 — The association analyses result from simple GEE model (obesity as dependent variable). [file 1471-2458-13-989-S2.doc]

**Table S1 The association analyses result from simple GEE model(obesity as dependent variable)**

| **Quartiles** | **estimate** | **ERR** | **Z** | **P>|Z|** | **RR** | **lower 95% Confidence Limits** | **upper 95% Confidence Limits** |
| --- | --- | --- | --- | --- | --- | --- | --- |
| **red blood cell** |  |  |  |  |  |  |  |
| **Q4** | 0.674 | 0.093 | 7.288 | <0.001 | 1.963 | 1.637 | 2.353 |
| **Q3** | 0.459 | 0.095 | 4.822 | <0.001 | 1.583 | 1.313 | 1.907 |
| **Q2** | 0.019 | 0.099 | 0.191 | 0.848 | 1.019 | 0.840 | 1.237 |
| **Q1** | ref | ref | ref | ref | ref | ref | ref |
| **hemoglobin** |  |  |  |  |  |  |  |
| **Q4** | 0.882 | 0.098 | 8.955 | <0.001 | 2.415 | 1.991 | 2.929 |
| **Q3** | 0.539 | 0.103 | 5.224 | <0.001 | 1.714 | 1.400 | 2.098 |
| **Q2** | 0.383 | 0.102 | 3.757 | <0.001 | 1.466 | 1.201 | 1.790 |
| **Q1** | ref | ref | ref | ref | ref | ref | ref |
| **hematocrit** |  |  |  |  |  |  |  |
| **Q4** | 0.657 | 0.096 | 6.824 | <0.001 | 1.928 | 1.597 | 2.328 |
| **Q3** | 0.447 | 0.099 | 4.495 | <0.001 | 1.563 | 1.287 | 1.900 |
| **Q2** | 0.253 | 0.098 | 2.585 | 0.010 | 1.288 | 1.063 | 1.560 |
| **Q1** | ref | ref | ref | ref | ref | ref | ref |
| **gender** | -0.745 | 0.062 | -12.021 | <0.001 | 0.475 | 0.420 | 0.536 |
| **age** | 0.337 | 0.017 | 19.527 | <0.001 | 1.401 | 1.354 | 1.449 |
| **GGT** | 0.010 | 0.001 | 6.938 | <0.001 | 1.010 | 1.007 | 1.012 |
| **ALB** | -0.071 | 0.011 | -6.178 | <0.001 | 0.932 | 0.911 | 0.953 |
| **GLO** | 0.040 | 0.007 | 5.611 | <0.001 | 1.041 | 1.026 | 1.056 |
| **BUN** | 0.076 | 0.026 | 2.917 | 0.004 | 1.079 | 1.025 | 1.136 |
| **S-Cr** | 0.011 | 0.004 | 2.680 | 0.007 | 1.011 | 1.003 | 1.018 |
| **WBC** | 0.178 | 0.019 | 9.403 | <0.001 | 1.194 | 1.151 | 1.239 |
| **PDW** | -0.018 | 0.020 | -0.870 | 0.384 | 0.982 | 0.944 | 1.022 |
| **MPV** | -0.074 | 0.043 | -1.739 | 0.082 | 0.929 | 0.854 | 1.009 |
| **PCT** | 0.002 | 0.175 | 0.013 | 0.990 | 1.002 | 0.712 | 1.411 |
| **diet** | 0.235 | 0.036 | 6.575 | <0.001 | 1.265 | 1.179 | 1.357 |
| **Drinking** | 0.120 | 0.023 | 5.118 | <0.001 | 1.128 | 1.077 | 1.181 |
| **smoking** | 0.079 | 0.026 | 3.041 | 0.002 | 1.083 | 1.029 | 1.140 |
| **sleep** | -0.039 | 0.042 | -0.907 | 0.364 | 0.962 | 0.885 | 1.046 |
| **exercise** | 0.001 | 0.080 | 0.008 | 0.994 | 1.001 | 0.856 | 1.169 |
