# Supplementary material for: Association between erythrocyte parameters and metabolic syndrome in urban Han Chinese: a longitudinal cohort study
Source: BMC Public Health. 2013 Oct 21;13:989. doi: 10.1186/1471-2458-13-989 (PMC4016498; doi:10.1186/1471-2458-13-989)
Supplement: Additional file 3: Table S2 — The association analyses result from simple GEE model (dyslipidemia as dependent variable). [file 1471-2458-13-989-S3.doc]

**Table S2 The association analyses result from simple GEE model(dyslipidemia as dependent variable)**

| **Quartiles** | **estimate** | **ERR** | **Z** | **P>|Z|** | **RR** | **lower 95% Confidence Limits** | **upper 95% Confidence Limits** |
| --- | --- | --- | --- | --- | --- | --- | --- |
| **red blood cell** |  |  |  |  |  |  |  |
| **Q4** | 0.747 | 0.068 | 11.032 | <0.001 | 2.110 | 1.848 | 2.410 |
| **Q3** | 0.423 | 0.072 | 5.893 | <0.001 | 1.527 | 1.326 | 1.758 |
| **Q2** | 0.184 | 0.074 | 2.504 | 0.012 | 1.202 | 1.041 | 1.389 |
| **Q1** | ref | ref | ref | ref | ref | ref | ref |
| **hemoglobin** |  |  |  |  |  |  |  |
| **Q4** | 0.812 | 0.064 | 12.601 | <0.001 | 2.252 | 1.985 | 2.555 |
| **Q3** | 0.392 | 0.069 | 5.664 | <0.001 | 1.480 | 1.292 | 1.695 |
| **Q2** | 0.108 | 0.071 | 1.511 | 0.131 | 1.114 | 0.968 | 1.281 |
| **Q1** | ref | ref | ref | ref | ref | ref | ref |
| **hematocrit** |  |  |  |  |  |  |  |
| **Q4** | 0.715 | 0.054 | 13.218 | <0.001 | 2.044 | 1.838 | 2.272 |
| **Q3** | 0.414 | 0.057 | 7.230 | <0.001 | 1.513 | 1.352 | 1.692 |
| **Q2** | 0.085 | 0.059 | 1.437 | 0.151 | 1.089 | 0.969 | 1.223 |
| **Q1** | ref | ref | ref | ref | ref | ref | ref |
| **gender** | -0.597 | 0.038 | -15.812 | <0.001 | 0.551 | 0.511 | 0.593 |
| **age** | 0.298 | 0.010 | 30.328 | <0.001 | 1.347 | 1.322 | 1.374 |
| **GGT** | 0.021 | 0.002 | 12.680 | <0.001 | 1.021 | 1.018 | 1.025 |
| **ALB** | 0.022 | 0.008 | 2.868 | 0.004 | 1.023 | 1.007 | 1.038 |
| **GLO** | 0.015 | 0.005 | 3.279 | 0.001 | 1.015 | 1.006 | 1.024 |
| **BUN** | 0.088 | 0.016 | 5.557 | <0.001 | 1.092 | 1.059 | 1.127 |
| **S-Cr** | 0.018 | 0.002 | 8.266 | <0.001 | 1.018 | 1.014 | 1.023 |
| **WBC** | 0.182 | 0.012 | 15.284 | <0.001 | 1.200 | 1.172 | 1.228 |
| **PDW** | -0.001 | 0.011 | -0.066 | 0.947 | 0.999 | 0.978 | 1.021 |
| **MPV** | -0.104 | 0.024 | -4.374 | <0.001 | 0.901 | 0.860 | 0.944 |
| **PCT** | 0.135 | 0.100 | 1.352 | 0.176 | 1.144 | 0.941 | 1.391 |
| **diet** | 0.207 | 0.021 | 9.722 | <0.001 | 1.231 | 1.180 | 1.283 |
| **Drinking** | 0.187 | 0.012 | 15.513 | <0.001 | 1.206 | 1.178 | 1.235 |
| **smoking** | 0.165 | 0.011 | 14.493 | <0.001 | 1.179 | 1.153 | 1.206 |
| **sleep** | 0.121 | 0.023 | 5.356 | <0.001 | 1.129 | 1.080 | 1.180 |
| **exercise** | -0.215 | 0.044 | -4.923 | <0.001 | 0.807 | 0.741 | 0.879 |
