# Supplementary material for: Association between erythrocyte parameters and metabolic syndrome in urban Han Chinese: a longitudinal cohort study
Source: BMC Public Health. 2013 Oct 21;13:989. doi: 10.1186/1471-2458-13-989 (PMC4016498; doi:10.1186/1471-2458-13-989)
Supplement: Additional file 4: Table S3 — The association analyses result from simple GEE model (hyperglycemia as dependent variable). [file 1471-2458-13-989-S4.doc]

**Table S3**  **The association analyses result from simple GEE model(hyperglycemia as dependent variable)**

| **Quartiles** | **estimate** | **ERR** | **Z** | **P>|Z|** | **RR** | **lower 95% Confidence Limits** | **upper 95% Confidence Limits** |
| --- | --- | --- | --- | --- | --- | --- | --- |
| **red blood cell** |  |  |  |  |  |  |  |
| **Q4** | 0.481 | 0.123 | 3.895 | <0.001 | 1.617 | 1.270 | 2.060 |
| **Q3** | 0.211 | 0.127 | 1.665 | 0.096 | 1.235 | 0.963 | 1.582 |
| **Q2** | 0.108 | 0.128 | 0.844 | 0.399 | 1.114 | 0.867 | 1.431 |
| **Q1** | ref | ref | ref | ref | ref | ref | ref |
| **hemoglobin** |  |  |  |  |  |  |  |
| **Q4** | 0.722 | 0.128 | 5.651 | <0.001 | 2.058 | 1.602 | 2.644 |
| **Q3** | 0.540 | 0.130 | 4.167 | <0.001 | 1.717 | 1.331 | 2.213 |
| **Q2** | 0.290 | 0.136 | 2.136 | 0.033 | 1.336 | 1.024 | 1.743 |
| **Q1** | ref | ref | ref | ref | ref | ref | ref |
| **hematocrit** |  |  |  |  |  |  |  |
| **Q4** | 0.364 | 0.124 | 2.937 | 0.003 | 1.440 | 1.129 | 1.836 |
| **Q3** | 0.333 | 0.123 | 2.709 | 0.007 | 1.395 | 1.096 | 1.774 |
| **Q2** | 0.056 | 0.129 | 0.438 | 0.662 | 1.058 | 0.822 | 1.361 |
| **Q1** | ref | ref | ref | ref | ref | ref | ref |
| **gender** | -0.497 | 0.081 | -6.114 | <0.001 | 0.608 | 0.519 | 0.713 |
| **age** | 0.547 | 0.023 | 23.799 | <0.001 | 1.727 | 1.651 | 1.807 |
| **GGT** | 0.009 | 0.001 | 6.775 | <0.001 | 1.009 | 1.006 | 1.012 |
| **ALB** | -0.065 | 0.015 | -4.256 | <0.001 | 0.937 | 0.910 | 0.966 |
| **GLO** | 0.073 | 0.009 | 8.561 | <0.001 | 1.076 | 1.058 | 1.094 |
| **BUN** | 0.128 | 0.032 | 3.980 | <0.001 | 1.137 | 1.067 | 1.210 |
| **S-Cr** | 0.008 | 0.004 | 2.209 | 0.027 | 1.008 | 1.001 | 1.015 |
| **WBC** | 0.166 | 0.024 | 7.031 | <0.001 | 1.181 | 1.128 | 1.237 |
| **PDW** | -0.038 | 0.025 | -1.502 | 0.133 | 0.963 | 0.916 | 1.012 |
| **MPV** | -0.102 | 0.053 | -1.927 | 0.054 | 0.903 | 0.815 | 1.002 |
| **PCT** | -0.008 | 0.076 | -0.106 | 0.915 | 0.992 | 0.854 | 1.152 |
| **diet** | 0.206 | 0.043 | 4.785 | <0.001 | 1.229 | 1.130 | 1.338 |
| **Drinking** | 0.025 | 0.029 | 0.839 | 0.401 | 1.025 | 0.968 | 1.086 |
| **smoking** | 0.086 | 0.025 | 3.377 | 0.001 | 1.089 | 1.037 | 1.145 |
| **sleep** | 0.025 | 0.044 | 0.561 | 0.575 | 1.025 | 0.940 | 1.119 |
| **exercise** | -0.090 | 0.091 | -0.981 | 0.327 | 0.914 | 0.764 | 1.094 |
