# Supplementary material for: Association between erythrocyte parameters and metabolic syndrome in urban Han Chinese: a longitudinal cohort study
Source: BMC Public Health. 2013 Oct 21;13:989. doi: 10.1186/1471-2458-13-989 (PMC4016498; doi:10.1186/1471-2458-13-989)
Supplement: Additional file 5: Table S4 — The association analyses result from simple GEE model (hypertension as dependent variable). [file 1471-2458-13-989-S5.doc]

**Table S4 The association analyses result from simple GEE model(hypertension as dependent variable)**

| **Quartiles** | **estimate** | **ERR** | **Z** | **P>|Z|** | **RR** | **lower 95% Confidence Limits** | **upper 95% Confidence Limits** |
| --- | --- | --- | --- | --- | --- | --- | --- |
| **red blood cell** |  |  |  |  |  |  |  |
| **Q4** | 0.709 | 0.118 | 6.007 | <0.001 | 2.031 | 1.612 | 2.560 |
| **Q3** | 0.390 | 0.122 | 3.207 | 0.001 | 1.478 | 1.164 | 1.876 |
| **Q2** | 0.158 | 0.129 | 1.224 | 0.221 | 1.172 | 0.909 | 1.509 |
| **Q1** | ref | ref | ref | ref | ref | ref | ref |
| **hemoglobin** |  |  |  |  |  |  |  |
| **Q4** | 0.882 | 0.117 | 7.507 | <0.001 | 2.416 | 1.919 | 3.041 |
| **Q3** | 0.453 | 0.124 | 3.660 | <0.001 | 1.572 | 1.234 | 2.004 |
| **Q2** | 0.301 | 0.130 | 2.303 | 0.021 | 1.351 | 1.046 | 1.744 |
| **Q1** | ref | ref | ref | ref | ref | ref | ref |
| **hematocrit** |  |  |  |  |  |  |  |
| **Q4** | 0.641 | 0.121 | 5.303 | <0.001 | 1.898 | 1.498 | 2.405 |
| **Q3** | 0.461 | 0.121 | 3.797 | <0.001 | 1.585 | 1.250 | 2.011 |
| **Q2** | 0.206 | 0.129 | 1.591 | 0.112 | 1.229 | 0.953 | 1.584 |
| **Q1** | ref | ref | ref | ref | ref | ref | ref |
| **gender** | -0.586 | 0.084 | -6.978 | <0.001 | 0.557 | 0.472 | 0.656 |
| **age** | 0.388 | 0.021 | 18.323 | <0.001 | 1.475 | 1.415 | 1.537 |
| **GGT** | 0.009 | 0.001 | 6.292 | <0.001 | 1.009 | 1.006 | 1.012 |
| **ALB** | -0.002 | 0.014 | -0.122 | 0.903 | 0.998 | 0.970 | 1.027 |
| **GLO** | 0.049 | 0.009 | 5.492 | <0.001 | 1.050 | 1.032 | 1.069 |
| **BUN** | 0.103 | 0.028 | 3.731 | <0.001 | 1.108 | 1.050 | 1.170 |
| **S-Cr** | 0.013 | 0.004 | 3.355 | 0.001 | 1.013 | 1.005 | 1.021 |
| **WBC** | 0.120 | 0.024 | 5.015 | <0.001 | 1.127 | 1.076 | 1.181 |
| **PDW** | 0.009 | 0.023 | 0.387 | 0.699 | 1.009 | 0.964 | 1.056 |
| **MPV** | 0.008 | 0.044 | 0.178 | 0.858 | 1.008 | 0.925 | 1.098 |
| **PCT** | -0.017 | 0.066 | -0.262 | 0.794 | 0.983 | 0.864 | 1.118 |
| **diet** | 0.113 | 0.044 | 2.547 | 0.011 | 1.119 | 1.026 | 1.221 |
| **Drinking** | 0.093 | 0.034 | 2.741 | 0.006 | 1.097 | 1.027 | 1.173 |
| **smoking** | 0.019 | 0.031 | 0.601 | 0.548 | 1.019 | 0.959 | 1.082 |
| **sleep** | 0.041 | 0.049 | 0.827 | 0.408 | 1.042 | 0.946 | 1.147 |
| **exercise** | 0.157 | 0.088 | 1.779 | 0.075 | 1.170 | 0.984 | 1.391 |
