# Supplementary material for: Association between erythrocyte parameters and metabolic syndrome in urban Han Chinese: a longitudinal cohort study
Source: BMC Public Health. 2013 Oct 21;13:989. doi: 10.1186/1471-2458-13-989 (PMC4016498; doi:10.1186/1471-2458-13-989)
Supplement: Additional file 6: Table S5 — Multiple GEE analysis of red blood cell and MetS after adjusting other potential confounding factors. [file 1471-2458-13-989-S6.doc]

**Table S5 Multiple GEE analysis of red blood cell and MetS after adjusting other potential confounding factors**

| **Quartiles** | **estimate** | **ERR** | **Z** | **P>|Z|** | **RR** | **lower 95% Confidence Limits** | **upper 95% Confidence Limits** |
| --- | --- | --- | --- | --- | --- | --- | --- |
| **red blood cell** |  |  |  |  |  |  |  |
| **Q4** | 1.104 | 0.348 | 3.171 | 0.002 | 3.016 | 1.525 | 5.967 |
| **Q3** | 0.523 | 0.300 | 1.742 | 0.081 | 1.688 | 0.937 | 3.040 |
| **Q2** | -0.039 | 0.300 | -0.131 | 0.895 | 0.961 | 0.534 | 1.730 |
| **Q1** | ref | ref | ref | ref | ref | ref | ref |
| **gender** | 0.142 | 0.297 | 0.478 | 0.633 | 1.152 | 0.644 | 2.062 |
| **age** | 0.030 | 0.008 | 3.617 | <0.001 | 1.030 | 1.014 | 1.047 |
| **GGT** | 0.012 | 0.002 | 6.720 | <0.001 | 1.012 | 1.008 | 1.015 |
| **ALB** | -0.054 | 0.032 | -1.655 | 0.098 | 0.948 | 0.889 | 1.010 |
| **GLO** | 0.075 | 0.018 | 4.181 | <0.001 | 1.078 | 1.041 | 1.117 |
| **BUN** | 0.116 | 0.057 | 2.028 | 0.043 | 1.123 | 1.004 | 1.256 |
| **WBC** | 0.227 | 0.041 | 5.558 | <0.001 | 1.255 | 1.158 | 1.359 |
| **diet** | 0.128 | 0.090 | 1.434 | 0.151 | 1.137 | 0.954 | 1.355 |
| **Drinking** | 0.039 | 0.066 | 0.590 | 0.555 | 1.040 | 0.913 | 1.185 |
| **smoking** | -0.001 | 0.058 | -0.023 | 0.982 | 0.999 | 0.892 | 1.119 |
