# Supplementary material for: Association between erythrocyte parameters and metabolic syndrome in urban Han Chinese: a longitudinal cohort study
Source: BMC Public Health. 2013 Oct 21;13:989. doi: 10.1186/1471-2458-13-989 (PMC4016498; doi:10.1186/1471-2458-13-989)
Supplement: Additional file 7: Table S6 — Multiple GEE analysis of red blood cell and obesity after adjusting other potential confounding factors. [file 1471-2458-13-989-S7.doc]

**Tab S6 Multiple GEE analysis of red blood cell and obesity after adjusting other potential confounding factors**

| **Quartiles** | **estimate** | **ERR** | **Z** | **P>|Z|** | **RR** | **lower 95% Confidence Limits** | **upper 95% Confidence Limits** |
| --- | --- | --- | --- | --- | --- | --- | --- |
| **red blood cell** |  |  |  |  |  |  |  |
| **Q4** | 0.474 | 0.123 | 3.847 | <0.001 | 1.606 | 1.262 | 2.045 |
| **Q3** | 0.364 | 0.107 | 3.392 | 0.001 | 1.439 | 1.166 | 1.775 |
| **Q2** | 0.006 | 0.102 | 0.057 | 0.955 | 1.006 | 0.823 | 1.229 |
| **Q1** | ref | ref | ref | ref | ref | ref | ref |
| **gender** | -0.104 | 0.132 | -0.789 | 0.430 | 0.901 | 0.696 | 1.167 |
| **age** | -0.002 | 0.003 | -0.742 | 0.458 | 0.998 | 0.991 | 1.004 |
| **GGT** | 0.010 | 0.002 | 5.543 | <0.001 | 1.010 | 1.007 | 1.014 |
| **ALB** | -0.096 | 0.013 | -7.605 | <0.001 | 0.909 | 0.886 | 0.931 |
| **GLO** | 0.031 | 0.008 | 4.012 | <0.001 | 1.031 | 1.016 | 1.047 |
| **BUN** | 0.046 | 0.030 | 1.532 | 0.126 | 1.047 | 0.987 | 1.111 |
| **S-Cr** | 0.006 | 0.005 | 1.317 | 0.188 | 1.006 | 0.997 | 1.015 |
| **WBC** | 0.133 | 0.020 | 6.515 | <0.001 | 1.142 | 1.097 | 1.189 |
| **diet** | 0.176 | 0.038 | 4.651 | <0.001 | 1.193 | 1.107 | 1.285 |
| **Drinking** | 0.051 | 0.029 | 1.773 | 0.076 | 1.053 | 0.995 | 1.114 |
| **smoking** | -0.018 | 0.029 | -0.609 | 0.542 | 0.982 | 0.927 | 1.041 |
