# Supplementary material for: Association between erythrocyte parameters and metabolic syndrome in urban Han Chinese: a longitudinal cohort study
Source: BMC Public Health. 2013 Oct 21;13:989. doi: 10.1186/1471-2458-13-989 (PMC4016498; doi:10.1186/1471-2458-13-989)
Supplement: Additional file 8: Table S7 — Multiple GEE analysis of red blood cell and hypertension after adjusting other potential confounding factors. [file 1471-2458-13-989-S8.doc]

**Table S7 Multiple GEE analysis of red blood cell and hypertension after adjusting other potential confounding factors**

| **Quartiles** | **estimate** | **ERR** | **Z** | **P>|Z|** | **RR** | **lower 95% Confidence Limits** | **upper 95% Confidence Limits** |
| --- | --- | --- | --- | --- | --- | --- | --- |
| **red blood cell** |  |  |  |  |  |  |  |
| **Q4** | 0.484 | 0.149 | 3.242 | 0.001 | 1.622 | 1.211 | 2.174 |
| **Q3** | 0.271 | 0.133 | 2.035 | 0.042 | 1.312 | 1.010 | 1.703 |
| **Q2** | 0.113 | 0.131 | 0.861 | 0.389 | 1.120 | 0.865 | 1.449 |
| **Q1** | ref | ref | ref | ref | ref | ref | ref |
| **gender** | -0.046 | 0.144 | -0.322 | 0.748 | 0.955 | 0.720 | 1.266 |
| **age** | 0.059 | 0.003 | 17.230 | <0.001 | 1.061 | 1.054 | 1.068 |
| **GGT** | 0.010 | 0.002 | 6.479 | <0.001 | 1.010 | 1.007 | 1.013 |
| **GLO** | 0.056 | 0.009 | 6.087 | <0.001 | 1.058 | 1.039 | 1.077 |
| **BUN** | 0.061 | 0.037 | 1.670 | 0.095 | 1.063 | 0.989 | 1.142 |
| **S-Cr** | 0.005 | 0.004 | 1.114 | 0.265 | 1.005 | 0.997 | 1.013 |
| **WBC** | 0.073 | 0.027 | 2.716 | 0.007 | 1.076 | 1.021 | 1.134 |
| **diet** | 0.061 | 0.049 | 1.246 | 0.213 | 1.062 | 0.966 | 1.169 |
| **Drinking** | 0.022 | 0.043 | 0.504 | 0.615 | 1.022 | 0.939 | 1.112 |
