# Supplementary material for: Association between erythrocyte parameters and metabolic syndrome in urban Han Chinese: a longitudinal cohort study
Source: BMC Public Health. 2013 Oct 21;13:989. doi: 10.1186/1471-2458-13-989 (PMC4016498; doi:10.1186/1471-2458-13-989)
Supplement: Additional file 9: Table S8 — Multiple GEE analysis of red blood cell and dyslipidemia after adjusting other potential confounding factors. [file 1471-2458-13-989-S9.doc]

**Table S8 Multiple GEE analysis of red blood cell and dyslipidemia after adjusting other potential confounding factors**

| **Quartiles** | **estimate** | **ERR** | **Z** | **P>|Z|** | **RR** | **lower 95% Confidence Limits** | **upper 95% Confidence Limits** |
| --- | --- | --- | --- | --- | --- | --- | --- |
| **red blood cell** |  |  |  |  |  |  |  |
| **Q4** | 0.431 | 0.089 | 4.824 | <0.001 | 1.539 | 1.292 | 1.833 |
| **Q3** | 0.251 | 0.079 | 3.173 | 0.002 | 1.285 | 1.101 | 1.500 |
| **Q2** | 0.123 | 0.075 | 1.632 | 0.103 | 1.131 | 0.976 | 1.311 |
| **Q1** | ref | ref | ref | ref | ref | ref | ref |
| **gender** | -0.035 | 0.082 | -0.432 | 0.666 | 0.965 | 0.822 | 1.133 |
| **age** | -0.013 | 0.003 | -4.920 | <0.001 | 0.987 | 0.982 | 0.992 |
| **GGT** | 0.015 | 0.002 | 8.015 | <0.001 | 1.015 | 1.011 | 1.019 |
| **ALB** | -0.038 | 0.010 | -3.785 | <0.001 | 0.963 | 0.944 | 0.982 |
| **GLO** | 0.040 | 0.006 | 7.045 | <0.001 | 1.041 | 1.029 | 1.052 |
| **BUN** | -0.027 | 0.023 | -1.187 | 0.235 | 0.973 | 0.930 | 1.018 |
| **S-Cr** | 0.007 | 0.002 | 3.276 | 0.001 | 1.007 | 1.003 | 1.011 |
| **WBC** | 0.132 | 0.015 | 8.819 | <0.001 | 1.141 | 1.108 | 1.175 |
| **MPV** | -0.070 | 0.030 | -2.347 | 0.019 | 0.932 | 0.880 | 0.989 |
| **diet** | 0.099 | 0.026 | 3.756 | <0.001 | 1.104 | 1.049 | 1.163 |
| **Drinking** | 0.043 | 0.019 | 2.286 | 0.022 | 1.044 | 1.006 | 1.084 |
| **smoking** | 0.061 | 0.018 | 3.505 | <0.001 | 1.063 | 1.027 | 1.100 |
| **sleep** | 0.141 | 0.030 | 4.703 | <0.001 | 1.151 | 1.086 | 1.221 |
| **exercise** | -0.306 | 0.055 | -5.609 | <0.001 | 0.736 | 0.661 | 0.819 |
