# Supplementary material for: Association between erythrocyte parameters and metabolic syndrome in urban Han Chinese: a longitudinal cohort study
Source: BMC Public Health. 2013 Oct 21;13:989. doi: 10.1186/1471-2458-13-989 (PMC4016498; doi:10.1186/1471-2458-13-989)
Supplement: Additional file 10: Table S9 — Multiple GEE analysis of red blood cell and hyperglycemia after adjusting other potential confounding factors. [file 1471-2458-13-989-S10.doc]

**Table S9 Multiple GEE analysis of red blood cell and hyperglycemia after adjusting other potential confounding factors**

| **Quartiles** | **estimate** | **ERR** | **Z** | **P>|Z|** | **RR** | **lower 95% Confidence Limits** | **upper 95% Confidence Limits** |
| --- | --- | --- | --- | --- | --- | --- | --- |
| **red blood cell** |  |  |  |  |  |  |  |
| **Q4** | 0.215 | 0.168 | 1.275 | 0.202 | 1.239 | 0.891 | 1.724 |
| **Q3** | 0.075 | 0.145 | 0.516 | 0.606 | 1.078 | 0.811 | 1.431 |
| **Q2** | 0.068 | 0.132 | 0.517 | 0.605 | 1.071 | 0.827 | 1.386 |
| **Q1** | ref | ref | ref | ref | ref | ref | ref |
| **gender** | -0.189 | 0.150 | -1.265 | 0.206 | 0.827 | 0.617 | 1.110 |
| **age** | 0.005 | 0.005 | 1.006 | 0.315 | 1.005 | 0.995 | 1.014 |
| **GGT** | 0.008 | 0.002 | 5.109 | <0.001 | 1.008 | 1.005 | 1.012 |
| **ALB** | -0.054 | 0.018 | -2.988 | 0.003 | 0.947 | 0.914 | 0.982 |
| **GLO** | 0.066 | 0.010 | 6.725 | <0.001 | 1.068 | 1.048 | 1.089 |
| **BUN** | 0.089 | 0.041 | 2.202 | 0.028 | 1.093 | 1.010 | 1.184 |
| **S-Cr** | 0.003 | 0.004 | 0.774 | 0.439 | 1.003 | 0.995 | 1.011 |
| **WBC** | 0.115 | 0.026 | 4.358 | <0.001 | 1.121 | 1.065 | 1.181 |
| **diet** | 0.147 | 0.047 | 3.119 | 0.002 | 1.158 | 1.056 | 1.271 |
| **smoking** | 0.020 | 0.030 | 0.688 | 0.492 | 1.021 | 0.963 | 1.082 |
