# Supplementary material for: Association between erythrocyte parameters and metabolic syndrome in urban Han Chinese: a longitudinal cohort study
Source: BMC Public Health. 2013 Oct 21;13:989. doi: 10.1186/1471-2458-13-989 (PMC4016498; doi:10.1186/1471-2458-13-989)
Supplement: Additional file 11: Table S10 — Multiple GEE analysis of hemoglobin and MetS after adjusting other potential confounding factors. [file 1471-2458-13-989-S11.doc]

**Table S10 Multiple GEE analysis of hemoglobin and MetS after adjusting other potential confounding factors**

| **Quartiles** | **estimate** | **ERR** | **Z** | **P>|Z|** | **RR** | **lower 95% Confidence Limits** | **upper 95% Confidence Limits** |
| --- | --- | --- | --- | --- | --- | --- | --- |
| **hemoglobin** |  |  |  |  |  |  |  |
| **Q4** | 1.101 | 0.361 | 3.047 | 0.002 | 3.008 | 1.481 | 6.109 |
| **Q3** | 0.454 | 0.311 | 1.460 | 0.144 | 1.575 | 0.856 | 2.898 |
| **Q2** | 0.340 | 0.243 | 1.398 | 0.162 | 1.405 | 0.872 | 2.264 |
| **Q1** | ref | ref | ref | ref | ref | ref | ref |
| **gender** | 0.012 | 0.311 | 0.040 | 0.968 | 1.012 | 0.551 | 1.861 |
| **age** | 0.015 | 0.007 | 2.018 | 0.044 | 1.015 | 1.000 | 1.030 |
| **GGT** | 0.011 | 0.002 | 7.108 | <0.001 | 1.011 | 1.008 | 1.014 |
| **ALB** | -0.073 | 0.027 | -2.660 | 0.008 | 0.930 | 0.882 | 0.981 |
| **GLO** | 0.062 | 0.016 | 3.779 | <0.001 | 1.063 | 1.030 | 1.098 |
| **BUN** | 0.154 | 0.049 | 3.134 | 0.002 | 1.166 | 1.059 | 1.284 |
| **WBC** | 0.219 | 0.035 | 6.340 | <0.001 | 1.245 | 1.164 | 1.332 |
| **diet** | 0.133 | 0.076 | 1.744 | 0.081 | 1.142 | 0.984 | 1.325 |
| **Drinking** | 0.035 | 0.054 | 0.646 | 0.518 | 1.035 | 0.932 | 1.150 |
| **smoking** | 0.002 | 0.047 | 0.040 | 0.968 | 1.002 | 0.914 | 1.098 |
