# Supplementary material for: Association between erythrocyte parameters and metabolic syndrome in urban Han Chinese: a longitudinal cohort study
Source: BMC Public Health. 2013 Oct 21;13:989. doi: 10.1186/1471-2458-13-989 (PMC4016498; doi:10.1186/1471-2458-13-989)
Supplement: Additional file 12: Table S11 — Multiple GEE analysis of hemoglobin and obesity after adjusting other potential confounding factors. [file 1471-2458-13-989-S12.doc]

**Table S11 Multiple GEE analysis of hemoglobin and obesity after adjusting other potential confounding factors**

| **Quartiles** | **estimate** | **ERR** | **Z** | **P>|Z|** | **RR** | **lower 95% Confidence Limits** | **upper 95% Confidence Limits** |
| --- | --- | --- | --- | --- | --- | --- | --- |
| **hemoglobin** |  |  |  |  |  |  |  |
| **Q4** | 0.693 | 0.148 | 4.690 | <0.001 | 2.000 | 1.497 | 2.673 |
| **Q3** | 0.447 | 0.125 | 3.574 | <0.001 | 1.564 | 1.224 | 1.998 |
| **Q2** | 0.387 | 0.102 | 3.788 | <0.001 | 1.473 | 1.205 | 1.800 |
| **Q1** | ref | ref | ref | ref | ref | ref | ref |
| **gender** | -0.049 | 0.140 | -0.351 | 0.725 | 0.952 | 0.724 | 1.252 |
| **age** | -0.003 | 0.003 | -1.030 | 0.303 | 0.997 | 0.991 | 1.003 |
| **GGT** | 0.010 | 0.002 | 5.399 | <0.001 | 1.010 | 1.006 | 1.014 |
| **ALB** | -0.099 | 0.013 | -7.807 | <0.001 | 0.905 | 0.883 | 0.928 |
| **GLO** | 0.030 | 0.008 | 3.829 | <0.001 | 1.030 | 1.015 | 1.046 |
| **BUN** | 0.048 | 0.030 | 1.589 | 0.112 | 1.049 | 0.989 | 1.113 |
| **S-Cr** | 0.006 | 0.004 | 1.335 | 0.182 | 1.006 | 0.997 | 1.015 |
| **WBC** | 0.136 | 0.020 | 6.762 | <0.001 | 1.146 | 1.102 | 1.192 |
| **diet** | 0.173 | 0.038 | 4.541 | <0.001 | 1.189 | 1.103 | 1.281 |
| **Drinking** | 0.045 | 0.029 | 1.567 | 0.117 | 1.046 | 0.989 | 1.107 |
| **smoking** | -0.027 | 0.029 | -0.915 | 0.360 | 0.974 | 0.919 | 1.031 |
