# Supplementary material for: Association between erythrocyte parameters and metabolic syndrome in urban Han Chinese: a longitudinal cohort study
Source: BMC Public Health. 2013 Oct 21;13:989. doi: 10.1186/1471-2458-13-989 (PMC4016498; doi:10.1186/1471-2458-13-989)
Supplement: Additional file 13: Table S12 — Multiple GEE analysis of hemoglobin and hypertension after adjusting other potential confounding factors. [file 1471-2458-13-989-S13.doc]

**Table S12 Multiple GEE analysis of hemoglobin and hypertension after adjusting other potential confounding factors**

| **Quartiles** | **estimate** | **ERR** | **Z** | **P>|Z|** | **RR** | **lower 95% Confidence Limits** | **upper 95% Confidence Limits** |
| --- | --- | --- | --- | --- | --- | --- | --- |
| **hemoglobin** |  |  |  |  |  |  |  |
| **Q4** | 0.749 | 0.166 | 4.500 | <0.001 | 2.114 | 1.526 | 2.929 |
| **Q3** | 0.390 | 0.143 | 2.724 | 0.006 | 1.477 | 1.116 | 1.956 |
| **Q2** | 0.273 | 0.129 | 2.108 | 0.035 | 1.314 | 1.019 | 1.693 |
| **Q1** | ref | ref | ref | ref | ref | ref | ref |
| **gender** | 0.104 | 0.157 | 0.659 | 0.510 | 1.109 | 0.815 | 1.510 |
| **age** | 0.059 | 0.003 | 17.466 | <0.001 | 1.061 | 1.054 | 1.068 |
| **GGT** | 0.010 | 0.002 | 6.288 | <0.001 | 1.010 | 1.007 | 1.013 |
| **GLO** | 0.056 | 0.009 | 6.005 | <0.001 | 1.057 | 1.038 | 1.077 |
| **BUN** | 0.065 | 0.037 | 1.777 | 0.076 | 1.067 | 0.993 | 1.146 |
| **S-Cr** | 0.004 | 0.004 | 1.151 | 0.250 | 1.004 | 0.997 | 1.012 |
| **WBC** | 0.074 | 0.027 | 2.755 | 0.006 | 1.076 | 1.021 | 1.134 |
| **diet** | 0.058 | 0.049 | 1.202 | 0.229 | 1.060 | 0.964 | 1.166 |
| **Drinking** | 0.012 | 0.043 | 0.277 | 0.782 | 1.012 | 0.930 | 1.102 |
