# Supplementary material for: Association between erythrocyte parameters and metabolic syndrome in urban Han Chinese: a longitudinal cohort study
Source: BMC Public Health. 2013 Oct 21;13:989. doi: 10.1186/1471-2458-13-989 (PMC4016498; doi:10.1186/1471-2458-13-989)
Supplement: Additional file 14: Table S13 — Multiple GEE analysis of hemoglobin and dyslipidemia after adjusting other potential confounding factors. [file 1471-2458-13-989-S14.doc]

**Table S13 Multiple GEE analysis of hemoglobin and dyslipidemia after adjusting other potential confounding factors**

| **Quartiles** | **estimate** | **ERR** | **Z** | **P>|Z|** | **RR** | **lower 95% Confidence Limits** | **upper 95% Confidence Limits** |
| --- | --- | --- | --- | --- | --- | --- | --- |
| **hemoglobin** |  |  |  |  |  |  |  |
| **Q4** | 0.536 | 0.102 | 5.265 | <0.001 | 1.709 | 1.400 | 2.087 |
| **Q3** | 0.254 | 0.084 | 3.006 | 0.003 | 1.289 | 1.092 | 1.521 |
| **Q2** | 0.082 | 0.071 | 1.149 | 0.251 | 1.086 | 0.944 | 1.249 |
| **Q1** | ref | ref | ref | ref | ref | ref | ref |
| **gender** | 0.062 | 0.092 | 0.674 | 0.501 | 1.064 | 0.888 | 1.275 |
| **age** | -0.013 | 0.003 | -5.200 | <0.001 | 0.987 | 0.982 | 0.992 |
| **GGT** | 0.015 | 0.002 | 7.905 | <0.001 | 1.015 | 1.011 | 1.019 |
| **ALB** | -0.038 | 0.010 | -3.821 | <0.001 | 0.962 | 0.944 | 0.981 |
| **GLO** | 0.040 | 0.006 | 6.998 | <0.001 | 1.041 | 1.029 | 1.052 |
| **BUN** | -0.027 | 0.023 | -1.193 | 0.233 | 0.973 | 0.930 | 1.018 |
| **S-Cr** | 0.007 | 0.002 | 3.315 | 0.001 | 1.007 | 1.003 | 1.011 |
| **WBC** | 0.135 | 0.015 | 9.074 | <0.001 | 1.144 | 1.111 | 1.178 |
| **MPV** | -0.061 | 0.030 | -2.078 | 0.038 | 0.941 | 0.888 | 0.997 |
| **diet** | 0.098 | 0.026 | 3.701 | <0.001 | 1.103 | 1.047 | 1.161 |
| **Drinking** | 0.039 | 0.019 | 2.024 | 0.043 | 1.039 | 1.001 | 1.079 |
| **smoking** | 0.052 | 0.017 | 3.002 | 0.003 | 1.054 | 1.018 | 1.090 |
| **sleep** | 0.139 | 0.030 | 4.656 | <0.001 | 1.150 | 1.084 | 1.219 |
| **exercise** | -0.310 | 0.055 | -5.673 | <0.001 | 0.733 | 0.659 | 0.816 |
