# Supplementary material for: Association between erythrocyte parameters and metabolic syndrome in urban Han Chinese: a longitudinal cohort study
Source: BMC Public Health. 2013 Oct 21;13:989. doi: 10.1186/1471-2458-13-989 (PMC4016498; doi:10.1186/1471-2458-13-989)
Supplement: Additional file 15: Table S14 — Multiple GEE analysis of hemoglobin and hyperglycemia after adjusting other potential confounding factors. [file 1471-2458-13-989-S15.doc]

**Table S14 Multiple GEE analysis of hemoglobin and hyperglycemia after adjusting other potential confounding factors**

| **Quartiles** | **estimate** | **ERR** | **z** | **P>|z|** | **RR** | **lower 95% Confidence Limits** | **upper 95% Confidence Limits** |
| --- | --- | --- | --- | --- | --- | --- | --- |
| **hemoglobin** |  |  |  |  |  |  |  |
| **Q4** | 0.654 | 0.191 | 3.427 | 0.001 | 1.923 | 1.323 | 2.796 |
| **Q3** | 0.519 | 0.159 | 3.270 | 0.001 | 1.680 | 1.231 | 2.292 |
| **Q2** | 0.307 | 0.137 | 2.239 | 0.025 | 1.359 | 1.039 | 1.777 |
| **Q1** | ref | ref | ref | ref | ref | ref | ref |
| **gender** | 0.043 | 0.163 | 0.264 | 0.792 | 1.044 | 0.759 | 1.435 |
| **age** | 0.004 | 0.005 | 0.880 | 0.379 | 1.004 | 0.995 | 1.014 |
| **GGT** | 0.008 | 0.002 | 4.988 | <0.001 | 1.008 | 1.005 | 1.011 |
| **ALB** | -0.064 | 0.018 | -3.548 | <0.001 | 0.938 | 0.905 | 0.972 |
| **GLO** | 0.065 | 0.010 | 6.487 | <0.001 | 1.067 | 1.046 | 1.088 |
| **BUN** | 0.094 | 0.041 | 2.303 | 0.021 | 1.098 | 1.014 | 1.190 |
| **S-Cr** | 0.003 | 0.004 | 0.852 | 0.394 | 1.003 | 0.996 | 1.011 |
| **WBC** | 0.111 | 0.027 | 4.172 | <0.001 | 1.117 | 1.061 | 1.177 |
| **diet** | 0.144 | 0.047 | 3.059 | 0.002 | 1.155 | 1.053 | 1.267 |
| **smoking** | 0.011 | 0.030 | 0.379 | 0.705 | 1.011 | 0.954 | 1.071 |
