# Supplementary material for: Association between erythrocyte parameters and metabolic syndrome in urban Han Chinese: a longitudinal cohort study
Source: BMC Public Health. 2013 Oct 21;13:989. doi: 10.1186/1471-2458-13-989 (PMC4016498; doi:10.1186/1471-2458-13-989)
Supplement: Additional file 16: Table S15 — Multiple GEE analysis of hematocrit and MetS after adjusting other potential confounding factors. [file 1471-2458-13-989-S16.doc]

**Table S15 Multiple GEE analysis of hematocrit and MetS after adjusting other potential confounding factors**

| **Quartiles** | **estimate** | **ERR** | **Z** | **P>|Z|** | **RR** | **lower 95% Confidence Limits** | **upper 95% Confidence Limits** |
| --- | --- | --- | --- | --- | --- | --- | --- |
| **hematocrit** |  |  |  |  |  |  |  |
| **Q4** | 0.583 | 0.332 | 1.754 | 0.079 | 1.792 | 0.934 | 3.437 |
| **Q3** | 0.214 | 0.302 | 0.708 | 0.479 | 1.238 | 0.685 | 2.238 |
| **Q2** | 0.034 | 0.254 | 0.135 | 0.892 | 1.035 | 0.630 | 1.701 |
| **Q1** | ref | ref | ref | ref | ref | ref | ref |
| **gender** | -0.233 | 0.288 | -0.809 | 0.419 | 0.792 | 0.450 | 1.393 |
| **age** | 0.014 | 0.007 | 1.892 | 0.058 | 1.014 | 1.000 | 1.029 |
| **GGT** | 0.011 | 0.001 | 7.608 | <0.001 | 1.011 | 1.008 | 1.014 |
| **ALB** | -0.064 | 0.027 | -2.382 | 0.017 | 0.938 | 0.889 | 0.989 |
| **GLO** | 0.065 | 0.016 | 4.024 | <0.001 | 1.067 | 1.034 | 1.101 |
| **BUN** | 0.148 | 0.049 | 3.023 | 0.003 | 1.160 | 1.054 | 1.277 |
| **WBC** | 0.220 | 0.034 | 6.414 | <0.001 | 1.246 | 1.165 | 1.332 |
| **diet** | 0.136 | 0.077 | 1.767 | 0.077 | 1.145 | 0.985 | 1.331 |
| **Drinking** | 0.037 | 0.055 | 0.683 | 0.495 | 1.038 | 0.933 | 1.155 |
| **smoking** | 0.008 | 0.048 | 0.170 | 0.865 | 1.008 | 0.918 | 1.107 |
