# Supplementary material for: Association between erythrocyte parameters and metabolic syndrome in urban Han Chinese: a longitudinal cohort study
Source: BMC Public Health. 2013 Oct 21;13:989. doi: 10.1186/1471-2458-13-989 (PMC4016498; doi:10.1186/1471-2458-13-989)
Supplement: Additional file 17: Table S16 — Multiple GEE analysis of hematocrit and obesity after adjusting other potential confounding factors. [file 1471-2458-13-989-S17.doc]

**Table S16 Multiple GEE analysis of hematocrit and obesity after adjusting other potential confounding factors**

| **Quartiles** | **estimate** | **ERR** | **Z** | **P>|Z|** | **RR** | **lower 95% Confidence Limits** | **upper 95% Confidence Limits** |
| --- | --- | --- | --- | --- | --- | --- | --- |
| **hematocrit** |  |  |  |  |  |  |  |
| **Q4** | 0.362 | 0.138 | 2.616 | 0.009 | 1.436 | 1.095 | 1.884 |
| **Q3** | 0.297 | 0.119 | 2.496 | 0.013 | 1.346 | 1.066 | 1.701 |
| **Q2** | 0.220 | 0.100 | 2.197 | 0.028 | 1.246 | 1.024 | 1.515 |
| **Q1** | ref | ref | ref | ref | ref | ref | ref |
| **gender** | -0.203 | 0.137 | -1.484 | 0.138 | 0.816 | 0.624 | 1.067 |
| **age** | -0.004 | 0.003 | -1.330 | 0.184 | 0.996 | 0.990 | 1.002 |
| **GGT** | 0.010 | 0.002 | 5.492 | <0.001 | 1.010 | 1.006 | 1.014 |
| **ALB** | -0.093 | 0.013 | -7.366 | <0.001 | 0.911 | 0.889 | 0.934 |
| **GLO** | 0.032 | 0.008 | 4.188 | <0.001 | 1.033 | 1.017 | 1.048 |
| **BUN** | 0.045 | 0.030 | 1.479 | 0.139 | 1.046 | 0.986 | 1.110 |
| **S-Cr** | 0.006 | 0.005 | 1.295 | 0.195 | 1.006 | 0.997 | 1.016 |
| **WBC** | 0.137 | 0.020 | 6.808 | <0.001 | 1.147 | 1.103 | 1.193 |
| **diet** | 0.174 | 0.038 | 4.562 | <0.001 | 1.190 | 1.104 | 1.283 |
| **Drinking** | 0.048 | 0.029 | 1.654 | 0.098 | 1.049 | 0.991 | 1.110 |
| **smoking** | -0.023 | 0.029 | -0.782 | 0.434 | 0.977 | 0.922 | 1.035 |
