# Supplementary material for: Association between erythrocyte parameters and metabolic syndrome in urban Han Chinese: a longitudinal cohort study
Source: BMC Public Health. 2013 Oct 21;13:989. doi: 10.1186/1471-2458-13-989 (PMC4016498; doi:10.1186/1471-2458-13-989)
Supplement: Additional file 18: Table S17 — Multiple GEE analysis of hematocrit and hypertension after adjusting other potential confounding factors. [file 1471-2458-13-989-S18.doc]

**Table S17 Multiple GEE analysis of hematocrit and hypertension after adjusting other potential confounding factors**

| **Quartiles** | **estimate** | **ERR** | **Z** | **P>|Z|** | **RR** | **lower 95% Confidence Limits** | **upper 95% Confidence Limits** |
| --- | --- | --- | --- | --- | --- | --- | --- |
| **hematocrit** |  |  |  |  |  |  |  |
| **Q4** | 0.368 | 0.159 | 2.313 | 0.021 | 1.445 | 1.058 | 1.974 |
| **Q3** | 0.324 | 0.137 | 2.376 | 0.018 | 1.383 | 1.058 | 1.808 |
| **Q2** | 0.149 | 0.130 | 1.150 | 0.250 | 1.161 | 0.900 | 1.496 |
| **Q1** | ref | ref | ref | ref | ref | ref | ref |
| **gender** | -0.084 | 0.150 | -0.558 | 0.577 | 0.920 | 0.685 | 1.234 |
| **age** | 0.057 | 0.003 | 16.986 | <0.001 | 1.059 | 1.052 | 1.066 |
| **GGT** | 0.010 | 0.002 | 6.490 | <0.001 | 1.010 | 1.007 | 1.013 |
| **GLO** | 0.057 | 0.009 | 6.126 | <0.001 | 1.059 | 1.039 | 1.078 |
| **BUN** | 0.061 | 0.037 | 1.660 | 0.097 | 1.063 | 0.989 | 1.142 |
| **S-Cr** | 0.005 | 0.004 | 1.109 | 0.268 | 1.005 | 0.996 | 1.013 |
| **WBC** | 0.078 | 0.027 | 2.924 | 0.003 | 1.081 | 1.026 | 1.139 |
| **diet** | 0.059 | 0.049 | 1.204 | 0.229 | 1.061 | 0.964 | 1.167 |
| **Drinking** | 0.018 | 0.043 | 0.408 | 0.684 | 1.018 | 0.935 | 1.108 |
