# Supplementary material for: Association between erythrocyte parameters and metabolic syndrome in urban Han Chinese: a longitudinal cohort study
Source: BMC Public Health. 2013 Oct 21;13:989. doi: 10.1186/1471-2458-13-989 (PMC4016498; doi:10.1186/1471-2458-13-989)
Supplement: Additional file 19: Table S18 — Multiple GEE analysis of hematocrit and dyslipidemia after adjusting other potential confounding factors. [file 1471-2458-13-989-S19.doc]

**Table S18 Multiple GEE analysis of hematocrit and dyslipidemia after adjusting other potential confounding factors**

| **Quartiles** | **estimate** | **ERR** | **Z** | **P>|Z|** | **RR** | **lower 95% Confidence Limits** | **upper 95% Confidence Limits** |
| --- | --- | --- | --- | --- | --- | --- | --- |
| **hematocrit** |  |  |  |  |  |  |  |
| **Q4** | 0.257 | 0.098 | 2.623 | 0.009 | 1.293 | 1.067 | 1.567 |
| **Q3** | 0.195 | 0.083 | 2.356 | 0.018 | 1.215 | 1.033 | 1.430 |
| **Q2** | 0.003 | 0.071 | 0.039 | 0.969 | 1.003 | 0.872 | 1.153 |
| **Q1** | ref | ref | ref | ref | ref | ref | ref |
| **gender** | -0.083 | 0.087 | -0.950 | 0.342 | 0.921 | 0.776 | 1.092 |
| **age** | -0.014 | 0.003 | -5.492 | <0.001 | 0.986 | 0.981 | 0.991 |
| **GGT** | 0.015 | 0.002 | 8.009 | <0.001 | 1.015 | 1.011 | 1.019 |
| **ALB** | -0.034 | 0.010 | -3.368 | 0.001 | 0.966 | 0.947 | 0.986 |
| **GLO** | 0.041 | 0.006 | 7.254 | <0.001 | 1.042 | 1.030 | 1.054 |
| **BUN** | -0.028 | 0.023 | -1.210 | 0.226 | 0.973 | 0.930 | 1.017 |
| **S-Cr** | 0.007 | 0.002 | 3.235 | 0.001 | 1.007 | 1.003 | 1.011 |
| **WBC** | 0.136 | 0.015 | 9.146 | 0.000 | 1.146 | 1.113 | 1.180 |
| **MPV** | -0.065 | 0.030 | -2.189 | 0.029 | 0.937 | 0.884 | 0.993 |
| **diet** | 0.100 | 0.026 | 3.779 | <0.001 | 1.105 | 1.049 | 1.164 |
| **Drinking** | 0.041 | 0.019 | 2.149 | 0.032 | 1.042 | 1.004 | 1.082 |
| **smoking** | 0.055 | 0.017 | 3.135 | 0.002 | 1.056 | 1.021 | 1.093 |
| **sleep** | 0.140 | 0.030 | 4.693 | <0.001 | 1.151 | 1.085 | 1.220 |
| **exercise** | -0.308 | 0.055 | -5.641 | <0.001 | 0.735 | 0.660 | 0.818 |
