# Supplementary material for: Association between erythrocyte parameters and metabolic syndrome in urban Han Chinese: a longitudinal cohort study
Source: BMC Public Health. 2013 Oct 21;13:989. doi: 10.1186/1471-2458-13-989 (PMC4016498; doi:10.1186/1471-2458-13-989)
Supplement: Additional file 20: Table S19 — Multiple GEE analysis of hematocrit and hyperglycemia after adjusting other potential confounding factors. [file 1471-2458-13-989-S20.doc]

**Table S19 Multiple GEE analysis of hematocrit and hyperglycemia after adjusting other potential confounding factors**

| **Quartiles** | **estimate** | **ERR** | **Z** | **P>|Z|** | **RR** | **lower 95% Confidence Limits** | **upper 95% Confidence Limits** |
| --- | --- | --- | --- | --- | --- | --- | --- |
| **hematocrit** |  |  |  |  |  |  |  |
| **Q4** | 0.028 | 0.179 | 0.158 | 0.874 | 1.029 | 0.724 | 1.463 |
| **Q3** | 0.151 | 0.152 | 0.995 | 0.320 | 1.163 | 0.864 | 1.566 |
| **Q2** | 0.014 | 0.133 | 0.107 | 0.915 | 1.014 | 0.782 | 1.316 |
| **Q1** | ref | ref | ref | ref | ref | ref | ref |
| **gender** | -0.250 | 0.152 | -1.643 | 0.100 | 0.778 | 0.577 | 1.049 |
| **age** | 0.004 | 0.005 | 0.788 | 0.431 | 1.004 | 0.994 | 1.013 |
| **GGT** | 0.008 | 0.002 | 5.151 | <0.001 | 1.008 | 1.005 | 1.012 |
| **ALB** | -0.051 | 0.018 | -2.814 | 0.005 | 0.950 | 0.917 | 0.985 |
| **GLO** | 0.067 | 0.010 | 6.790 | <0.001 | 1.069 | 1.049 | 1.090 |
| **BUN** | 0.089 | 0.041 | 2.203 | 0.028 | 1.093 | 1.010 | 1.184 |
| **S-Cr** | 0.003 | 0.004 | 0.765 | 0.444 | 1.003 | 0.995 | 1.011 |
| **WBC** | 0.119 | 0.026 | 4.516 | <0.001 | 1.126 | 1.069 | 1.186 |
| **diet** | 0.147 | 0.047 | 3.110 | 0.002 | 1.158 | 1.056 | 1.270 |
| **smoking** | 0.019 | 0.030 | 0.627 | 0.531 | 1.019 | 0.961 | 1.080 |
